# The adduct of di-μ_2_-hydroxido-bis­[chlorido­di­phenyl­tin(IV)] with two mol­ecules of 2-vinyl­pyridine

**DOI:** 10.1107/S2056989026007152

**Published:** 2026-07-16

**Authors:** Tobias Gieschen, Hans Reuter

**Affiliations:** a Chemistry, Osnabrück University, Barabarstr. 7, 49069 Osnabrück, Germany; University of Buenos Aires, Argentina

**Keywords:** crystal structure, di­phenyl­tin(IV) dichloride, diorganotin(IV) hydroxide-halide, hydrolysis, 2-vinyl­pyridine, hydrogen bonding

## Abstract

The solid-state structure of the adduct of di­phenyl­tin(IV) hydroxide chloride, Ph_2_Sn(OH)Cl, with 2-vinyl­pyridine, ^2^Vipy, namely, di-μ-hydroxido-bis­[chlorido­diphenyl­tin(IV)]–2-ethenyl­pyridine (1/2), [Sn_2_(C_6_H_5_)_4_Cl_2_(OH)_2_]·2C_7_H_7_N, exhibits dimeric, hydrogen-bonded aggregates [Ph_2_Sn(OH)Cl·^2^Vipy]_2_. The aggregates are non-centrosymmetric but exhibit the characteristic structural features of Brønsted base, BB, stabilized diorganotin(IV) hydroxide-halides, [*R*_2_Sn(OH)Hal·BB]_2_, with trigonal–bipyramidally coordinated tin atoms and two bridging hydroxide groups. Non-centrosymmetry leads to a slightly bent and distorted rhombic, four-membered Sn_2_—O_2_ ring consisting of two different tin and oxygen atoms. As usual, Sn—O bond lengths depend on the position the hydroxyl groups adopt within the trigonal–bipyramidal coordination sphere of the Sn atoms [mean values: *d*(Sn—OH)_ax_ = 2.191 (9) Å, *d*(Sn—OH)_eq_ = 2.019 (3) Å]. Bond angles within the Sn_2_—O_2_ ring are acute [mean value: 71.1 (2)°] at the tin atoms and obtuse [mean value: 108.9 (3)°] at the oxygen atoms. Hydroxyl groups display a trigonal–planar constitution and are involved in hydrogen bonds [mean values: *d*(O⋯N) = 2.714 (8) Å; <(O—H⋯N) = 170 (5)°] to the N atoms of the 2-vinyl­pyridine mol­ecules.

## Abstract 2

Single crystals of [Ph_2_Sn(OH)Cl·^2^Vipy]_2_ obtained as side product during the reaction of di­phenyl­tin(IV) dichloride with 2-vinyl­pyridine, ^2^Vipy, have been structurally characterized by X-ray diffraction with respect to the influence of the adduct’s non-centrosymmetry on bond lengths and angles.

## 1. Chemical context

Diorganotin(IV) hydroxide halides, *R*_2_Sn(OH)Hal, are the first hydrolysis products of diorganotin(IV) dihalides, *R*_2_SnHal_2_. Although the hydrolysis products of the *dihalides* have been studied for a long time, only some few examples of *hydroxide halides* have been unambiguously characterized by X-ray diffraction. A complete set comprising all four halogens currently exists only for the bulky *t*-butyl group, *R* = ^*t*^Bu [Hal = F, α-Cl, Br (Puff *et al.*, 1985 ▸), Hal = β-Cl (Di Nicola *et al.*, 2011 ▸), and Hal = I (Reuter, 2022 ▸)]. These investigations revealed not only the dimeric nature of this class of compounds but also their general structural features with trigonal-bipyramidally coordinated tin atoms bridged via two hydroxide groups generating a four-membered Sn_2_O_2_ ring. Furthermore, there is only one additional example of a pure *hydroxide halide* described in the literature with *R* = *p*-tolyl and Hal = Br (Lo & Ng, 2009 ▸).

In the majority of cases, these primary hydrolysis products of diorganotin(IV) dihalides, in fact, undergo a condensation reaction giving rise to the formation of the more common tetra­organo-dihalogenido-distannoxanes, (*R*_2_SnHal)_2_O, which are in solution as well as in solid state dimeric, too, with a *ladder-type* Sn–O–Hal arrangement. In the literature, the structures of many *dihalogenido-distannoxanes* are described including those with *R* = Ph and Hal = Cl as pure substances (Vollano *et al.*, 1984 ▸) or as a CH_2_Cl_2_ solvate (Estudiante-Negrete *et al.*, 2004 ▸).

However, it is possible to isolate *hydroxide halides* – even if they normally tend to undergo condensation – when their hydroxyl groups are hindered to condensate because of the formation of hydrogen bonds to Brønsted bases, BB. Thus, for *R* = Ph and Hal = Cl this has been shown for BB = ethanol, EtOH, (Barba *et al.*, 2007 ▸) and quinoline, Quin, (Anacona *et al.*, 2003 ▸). The universality of this concept was underlined by [^*t*^Bu_2_Sn(OH)Cl]_2_·2DMSO, which was found in co-crystals with [(^*t*^Bu_2_Sn)_3_O(OH)_2_][I]_2_ ((Reuter & Wilberts, 2014 ▸), and by [Ph_2_Sn(OH)I]_2_·2DMPU (Reuter, 2025 ▸).
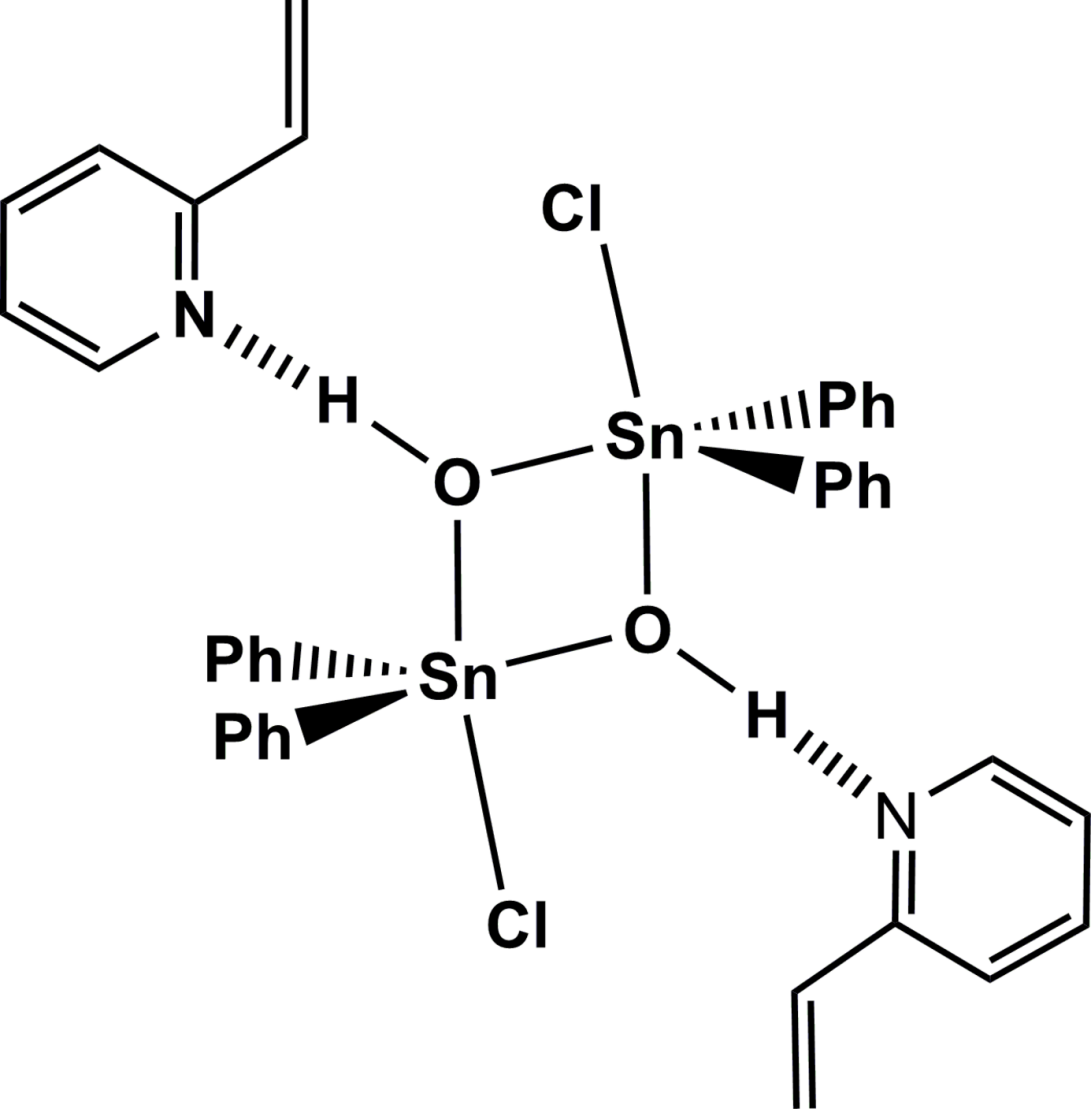


Here we present the first hydrogen-bond-stabilized *hydroxide halide* with a non-centrosymmetric, dimeric constitution obtained as side-product when we tried to synthesize a 1:2 complex of di­phenyl­tin(IV) dichloride, Ph_2_SnCl_2_, with 2-vinyl­pyridine, ^2^Vipy, in ethanol.

## 2. Structural commentary

**Figure 1:**
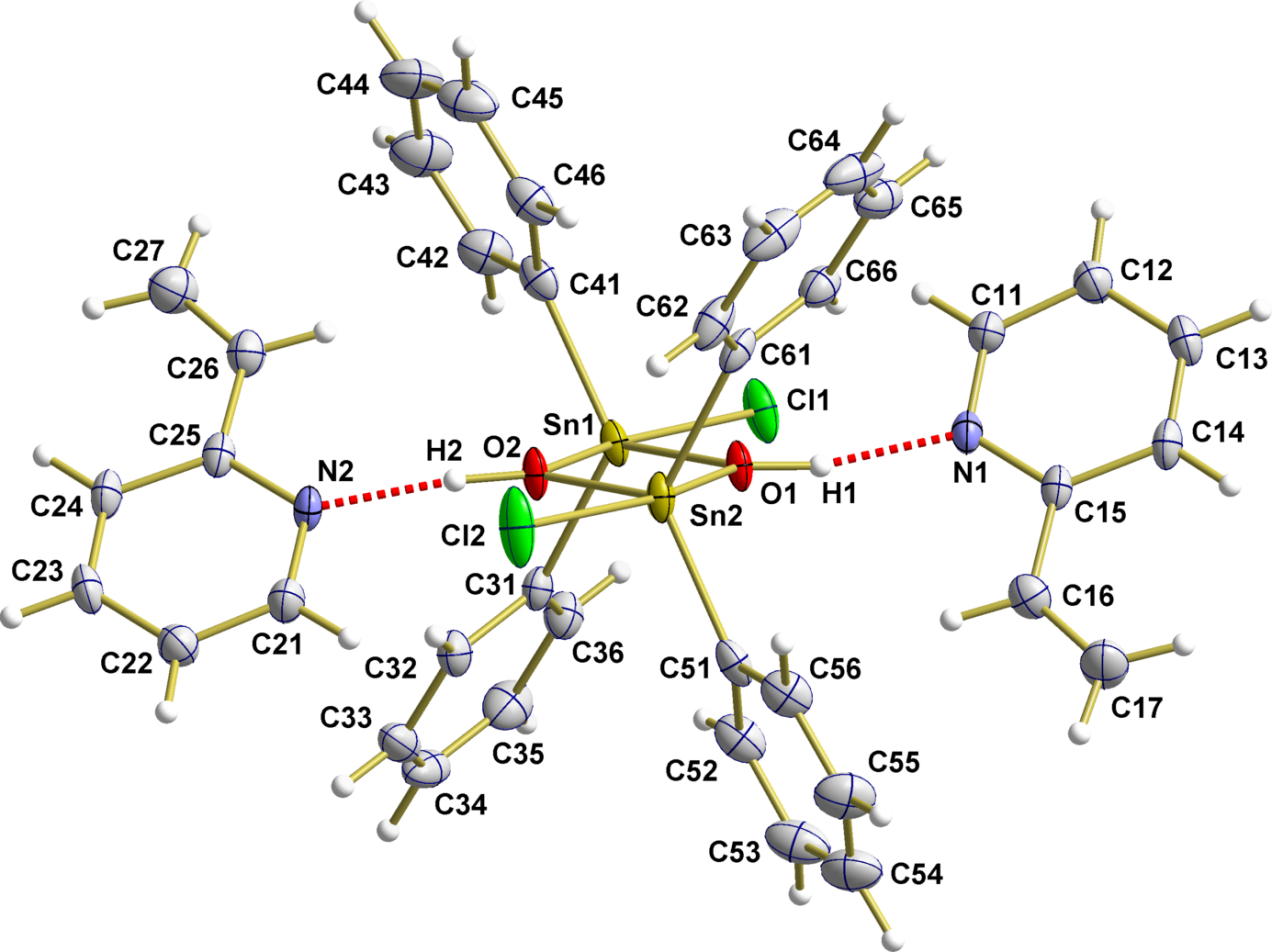
The asymmetric unit of the title compound shown with displacement ellipsoids at the 40% level. Red dashed lines represent the O—N⋯H hydrogen bonds.

**Figure 2:**
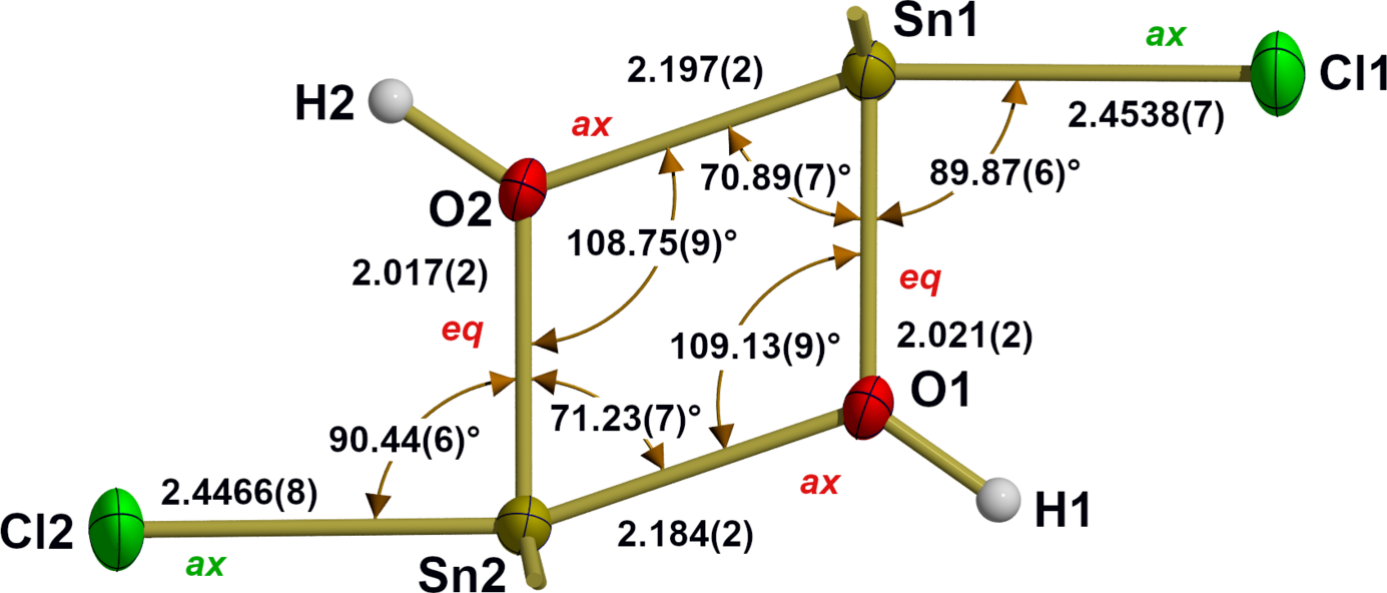
Representation of the four-membered Sn_2_O_2_ ring showing the most important bond angles (°) and bond lengths (Å). Positions of the oxygen and chlorine atoms within the trigonal–bipyramidal coordination of the tin atoms are labeled by use of the abbreviations *ax* (= axial) and *eq* (= equatorial). For clarity, Ph groups are stripped down to the Sn—C bonds drawn as shortened sticks.

The title compound crystallizes in the monoclinic space group *P*2_1_/*c* with four formula units, [Ph_2_Sn(OH)Cl·^2^Vipy]_2_ in the unit cell and one formula unit in the asymmetric unit with all atoms in general positions. The mol­ecule therefore belongs to point group *C*_1_ (Fig. 1 ▸) contrary to the *C_i_* symmetry of the corresponding phenyl compounds with ethanol and quinoline (Anacona *et al.*, 2003 ▸). As a result, the central, four-membered Sn_2_O_2_ ring is no longer planar but bent. The deviation from planarity, however, is very small as indicated by the dihedral angle between the two O–Sn–O planes of 0.38 (11)°. Apart from this non-planarity, the four-membered Sn_2_O_2_ ring (Fig. 2 ▸) exhibits the characteristic, slightly distorted, rhombic shape with acute angles at oxygen [70.89 (7)/71.23 (7)°] and obtuse ones at tin [109.13 (9)/108.75 (9)°], all in the range of the corresponding angles in the other *hydroxide-halides* (Puff *et al.*, 1985 ▸). The Sn—O bond lengths differ depending on whether the oxygen atom adopts an equatorial [2.0206 (19)/2.0172(19] Å] or an axial [2.1974 (18)/2.1845 (18) Å] position within the trigonal–bipyramidal coordination of the tin atoms.

The chlorine atoms adopt equatorial positions with a mean Sn–Cl distance of 2.450 (5) Å compared with the Sn—Cl distance of 2.45 (2) Å in the compound with quinoline. Somewhat longer Sn—Cl distances of 2.4748 (6) Å are found in the adduct with EtOH, and in the pure *t*-butyl compound with *d*(Sn—Cl)_mean_ = 2.506 (1) Å (Puff *et al.*, 1985 ▸), probably because the corresponding chlorine atoms are involved in hydrogen bonds.

The trigonal–bipyramidal coordination of the two crystallographically different tin atoms is completed by two phenyl groups in equatorial positions. Sn—C distances are in the range 2.112 (3)–2.128 (3) Å, mean value 2.120 (7) Å. By way of comparison: in the compound with quinoline and ethanol as BB, the Sn—C distances [BB = Quin: 2.120 (3)/2.134 (3) Å, 2.110 (4)/2.119 (3) Å; BB = EtOH: 2.114 (2)/2.120 (2) Å] are of comparable length. All of these values are significantly shorter from those found in the *t*-butyl compound [BB = DMSO: *d*(Sn—C) 2.193 (7) Å] underlining the observation that the Sn—C distances depend on the nature of the organic group.

C—C bond lengths within the four crystallographically different, almost planar phenyl rings range from 1.372 (6) to 1.401 (4) Å with a mean value of 1.386 (8) Å, which corresponds quite well with the value [1.387 (10) Å] given by Allen *et al.* (1987 ▸) for C—C bond lengths in phenyl groups. Among the bond angles within these phenyl rings, the value at the *ipso* carbon atom [mean value: 118.7 (1)°] is noteworthy because it is significantly smaller than in a regular hexa­gon, a phenomenon generally attributed to the *ipso-*effect (Domenicano *et al.*, 1983 ▸).

The structure of pure 2-vinyl­pyridine is not yet described in the literature. The compound has been used, however, as ligand in complexes with transition metals where it acts as Lewis base coordinating via the N atom of the pyridine moiety and/or as π-electron donor via its double bond. Typical examples of mononuclear complexes with 2-vinyl­pyridine as pure Lewis Base are [Os(^2^Vipy)_2_(^*i*^Pr_3_P)(CF_3_SO_3_), Fe*X*_2_(^2^Vipy)_2_ with *X* = *p*-tolyl­benzoate (Kuzelka *et al.*, 2003 ▸) or [Rh(^2^Vipy)_2_(COD)][CF_3_SO_3_] with COD = η^4^-cyclo­octa-1,5-diene (Beller *et al.*, 1999 ▸), and PdCl_2_(^2^Vipy)_2_ (Newkome *et al.*, 1988 ▸; Fronczek, 2015 ▸) while in complexes like RuCl(PPh_3_)(^2^Vipy)_2_ or RuCl_2_(PPh_3_)(^2^Vipy)·CH_2_Cl_2_ (Zhang *et al.*, 2007 ▸) both coordination (LB and π-donor) modes are realized. Compounds of 2-vinyl­pyridine acting as Brønsted Base with the N atom as hydrogen bond acceptor have not yet been described. In the title compound, the hydrogen bonds (Table 1 ▸) show donor–acceptor distances of 2.708 (3) and 2.720 (3) Å and bridging angles of 173.2/166.7°. In the quinoline adduct (Anacona *et al.*, 2003 ▸) the corresponding hydrogen bonds are somewhat longer [2.7564 (4)/2.787 (5)] and less linear [171 (3)°/163 (3)°].

The lack of information on the structure of the pure 2-vinyl­pyridine in combination with the multiple different binding modes in its transition metal complexes mentioned above, makes it difficult to evaluate the influence of the hydrogen-bond formation on the inter­nal bond lengths and angles of the almost planar 2-vinyl­pyridine mol­ecules of the title compound. Distortions of the two pyridine moieties from regular hexa­gons are expressed by N—C bond lengths of 1.330 (4) to 1.340 (4) Å [mean value: 1.336 (5) Å, reference value: 1.337 (12) Å for C_ar_—N_ar_ in pyridine (Allen *et al.*, 1987 ▸)], endocyclic bond angles at N of 118.1 (3)/118.0 (3)° accompanied by a widening [124.5 (3)/124.0 (3)°] of the bond angles at the carbon atoms C11/C21 in the *para* position to the vinyl group. The vinyl groups themselves are characterized by C—C single bond lengths of 1.469 (4)/1.471 (5) Å [reference value: 1.470 (15) for C*sp*^2^—C_ar_ in C=C—C_ar_, Allen *et al.*, 1987 ▸], C=C double bonds of 1.303 (5)/1.312 (5) Å [reference value: 1.339 (11) for C*sp*^2^=C*sp*^2^ in C=C—C_ar_, Allen *et al.*, 1987 ▸] and bond angles at the carbon atoms C16/C26 of 125.4 (3)/126.5 (3)°.

## 3. Supra­molecular features

**Figure 3:**
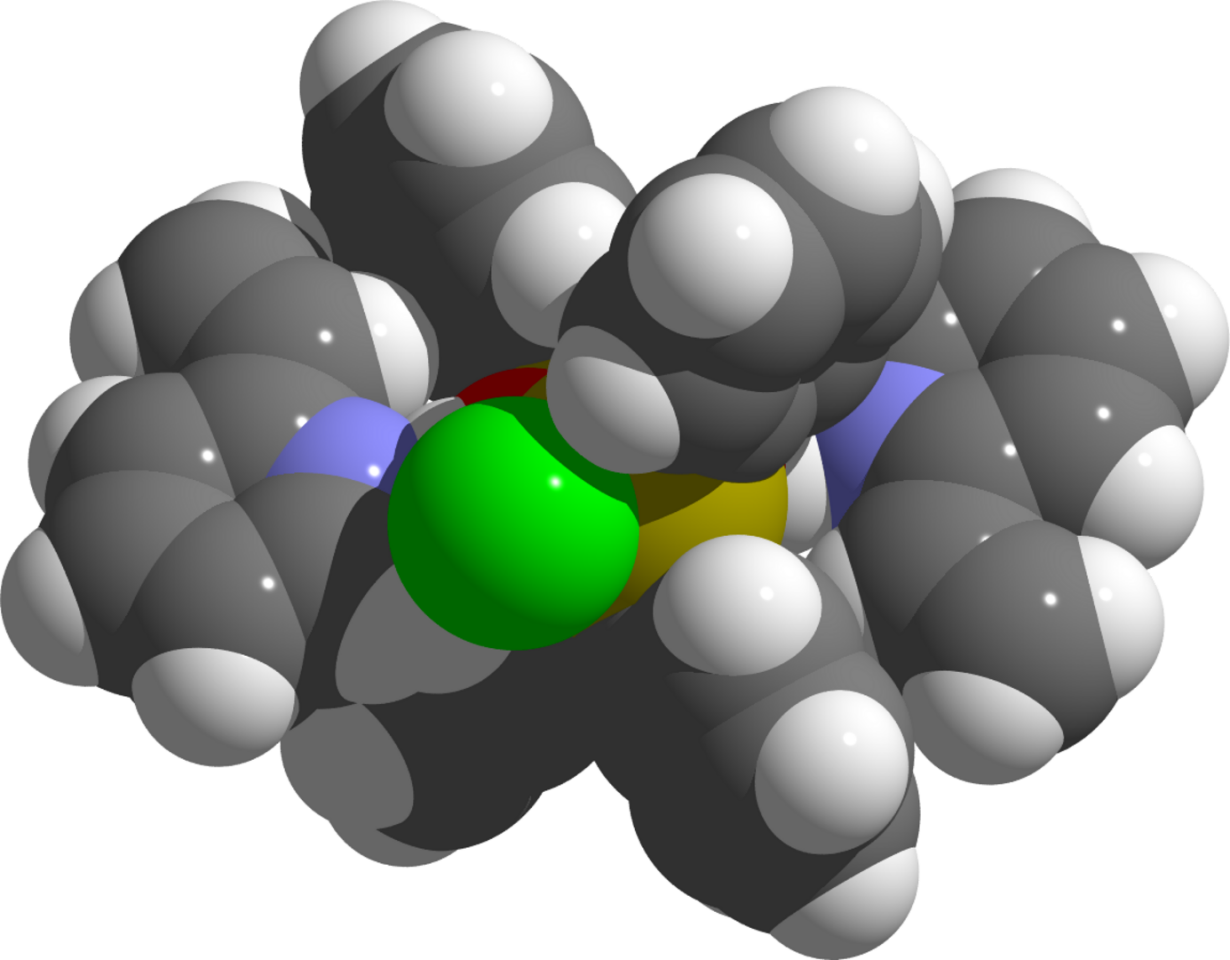
Space-filling model of the [Ph_2_Sn(OH)Cl·^2^Vipy]_2._ aggregates. Color code of the atoms: Cl = green, H = white, C = gray, O = red, Sn = gold, N = light blue.

With the formation of the hydrogen bonds to the 2-vinyl­pyridine mol­ecules the polar bonds of the [Ph_2_Sn(OH)Cl]_2_ mol­ecule are completely shielded except for the Sn—Cl bonds (Fig. 3 ▸). In contrast to the pure *t*-butyl compounds, ^*t*^Bu_2_Sn(OH)Hal, which exhibit one-dimensional chain structures due to OH⋯Hal hydrogen bonds, the title compound, like its quinoline counterpart, represents a mol­ecular structure. On the other hand, the EtOH adduct of Ph_2_Sn(OH)Cl constitutes a chain structure because the OH group of the ethanol mol­ecules acts as hydrogen donor to the chlorine atoms and as hydrogen acceptors to the hydroxyl groups.

**Figure 4:**
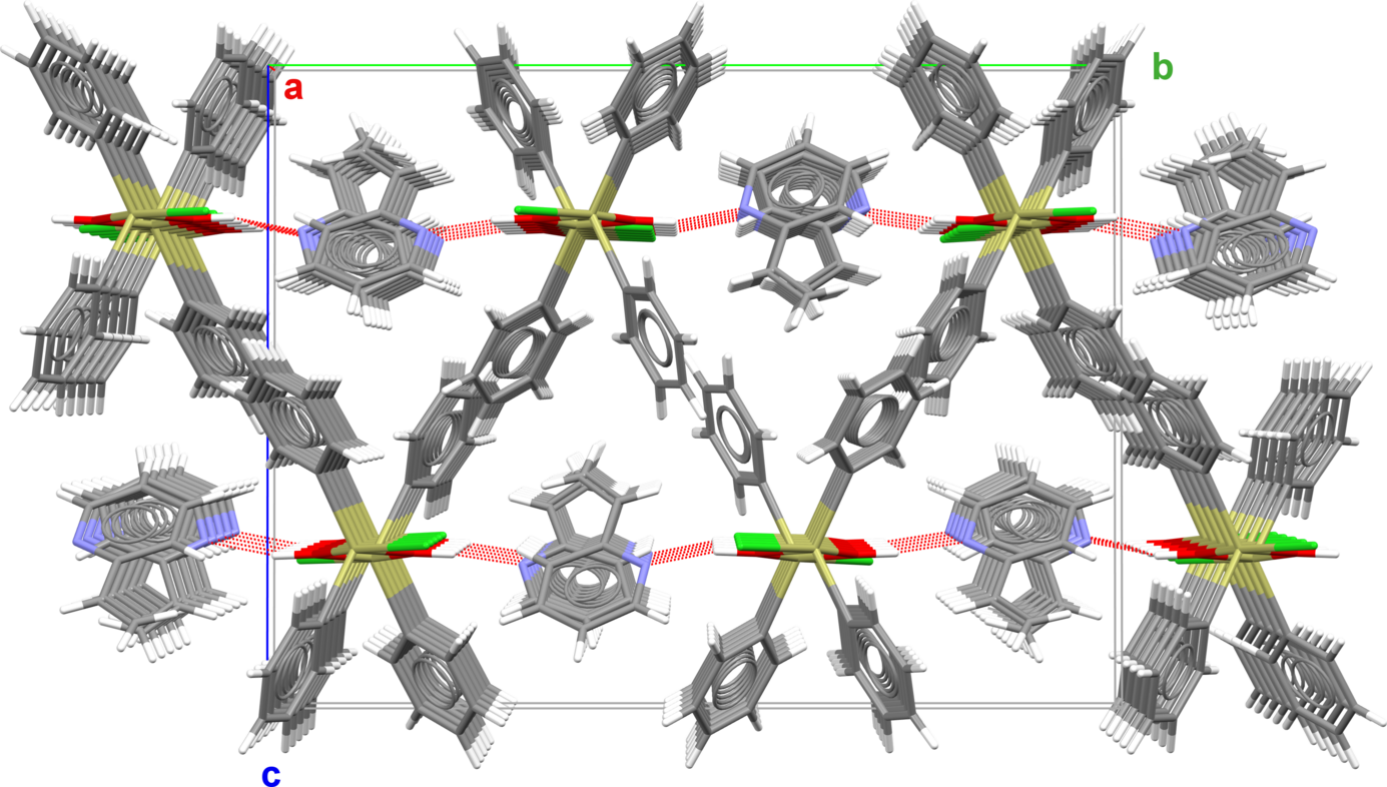
View of the three-dimensional network along the *bc* plane, highlighting the O—H⋯N hydrogen bonds.

Mol­ecules of the title compound are arranged in the solid state with their Sn—O planes in layers perpendicular to the *c*-axis probably because of weak inter­molecular inter­actions involving the Cl atoms with the hydrogen atoms of the organic moieties of neighboring mol­ecules (Fig. 4 ▸).

## 4. Synthesis and crystallization

The title compound was obtained as a side-product in a micro-scale experiment performed on a Petri dish screening the complex behavior of 2-vinyl­pyridine (Sigma Aldrich) towards di­phenyl­tin(IV) dichloride (Fluka). After prolonged standing in air, some colorless prisms suitable for X-ray diffraction could be isolated from the reaction mixture, probably as a result of partial hydrolysis of the dihalide.

## 5. Refinement

Crystal data, data collection and structure refinement details are summarized in Table 2 ▸. All H atoms were clearly identified in difference-Fourier syntheses. Those of the organic moieties were refined in idealized geometries and allowed to ride on their parent carbon atoms with 0.95 Å and common isotropic temperature factors for all hydrogen atoms of each organic moiety. The H atoms of the two hy­droxy groups were modeled with a common O—H distance of 0.96 Å before they were fixed and allowed to ride on the corresponding oxygen atom with one common isotropic temperature factor.

## Supplementary Material

Crystal structure: contains datablock(s) I. DOI: 10.1107/S2056989026007152/vu2020sup1.cif

Structure factors: contains datablock(s) I. DOI: 10.1107/S2056989026007152/vu2020Isup2.hkl

CCDC reference: 2572540

Additional supporting information:  crystallographic information; 3D view; checkCIF report

## Figures and Tables

**Table 1:** Hydrogen-bond geometry (Å, °)

*D*—H⋯*A*	*D*—H	H⋯*A*	*D*⋯*A*	*D*—H⋯*A*
O1—H1⋯N1	0.96	1.76	2.708 (3)	167
O2—H2⋯N2	0.96	1.76	2.720 (3)	173

**Table 2:** Experimental details

Crystal data	
Chemical formula	[Sn_2_(C_6_H_5_)_4_Cl_2_(OH)_2_]·2C_7_H_7_N
*M*_r_	860.97
Crystal system, space group	Monoclinic, *P*2_1_/*n*
Temperature (K)	100
*a*, *b*, *c* (Å)	10.4040 (4), 21.4649 (9), 16.2255 (7)
β (°)	94.746 (2)
*V* (Å^3^)	3611.1 (3)
*Z*	4
Radiation type	Mo *K*α
μ (mm^−1^)	1.57
Crystal size (mm)	0.31 × 0.17 × 0.09

Data collection	
Diffractometer	Bruker APEXII CCD
Absorption correction	Multi-scan (*SADABS*; Krause *et al.*, 2015 ▸)
*T*_min_, *T*_max_	0.718, 0.834
No. of measured, independent and observed [*I* > 2σ(*I*)] reflections	143749, 8729, 6258
*R*_int_	0.062
(sin θ/λ)_max_ (Å^−1^)	0.661

Refinement	
*R*[*F*^2^ > 2σ(*F*^2^)], *wR*(*F*^2^), *S*	0.031, 0.076, 1.04
No. of reflections	8729
No. of parameters	422
H-atom treatment	Only H-atom displacement parameters refined
Δρ_max_, Δρ_min_ (e Å^−3^)	1.21, −0.65

Computer programs: *APEX2* and *SAINT* (Bruker, 2009 ▸), *SHELXS97* (Sheldrick, 2008 ▸), *SHELXL2014/7* (Sheldrick, 2015 ▸), *DIAMOND* (Brandenburg, 2006 ▸), *Mercury* (Macrae *et al.*, 2020 ▸) and *publCIF* (Westrip, 2010 ▸).

## supplementary crystallographic information

### Di-µ-hydroxido-bis[chloridodiphenyltin(IV)]–2-ethenylpyridine (1/2) Crystal data

**Table:**

[Sn_2_(C_6_H_5_)_4_Cl_2_(OH)_2_]·2C_7_H_7_N	*F*(000) = 1712
*M**_r_* = 860.97	*D*_x_ = 1.584 Mg m^−^^3^
Monoclinic, *P*2_1_/*n*	Mo *K*α radiation, λ = 0.71073 Å
*a* = 10.4040 (4) Å	Cell parameters from 9965 reflections
*b* = 21.4649 (9) Å	θ = 2.3–27.4°
*c* = 16.2255 (7) Å	µ = 1.57 mm^−^^1^
β = 94.746 (2)°	*T* = 100 K
*V* = 3611.1 (3) Å^3^	Prism, colourless
*Z* = 4	0.31 × 0.17 × 0.09 mm

### Di-µ-hydroxido-bis[chloridodiphenyltin(IV)]–2-ethenylpyridine (1/2) Data collection

**Table:**

Bruker APEXII CCD diffractometer	6258 reflections with *I* > 2σ(*I*)
φ and ω scans	*R*_int_ = 0.062
Absorption correction: multi-scan (SADABS; Krause *et al.*, 2015)	θ_max_ = 28.0°, θ_min_ = 1.9°
*T*_min_ = 0.718, *T*_max_ = 0.834	*h* = −13→13
143749 measured reflections	*k* = −28→28
8729 independent reflections	*l* = −21→21

### Di-µ-hydroxido-bis[chloridodiphenyltin(IV)]–2-ethenylpyridine (1/2) Refinement

**Table:**

Refinement on *F*^2^	Primary atom site location: structure-invariant direct methods
Least-squares matrix: full	Hydrogen site location: mixed
*R*[*F*^2^ > 2σ(*F*^2^)] = 0.031	Only H-atom displacement parameters refined
*wR*(*F*^2^) = 0.076	*w* = 1/[σ^2^(*F*_o_^2^) + (0.0276*P*)^2^ + 4.3782*P*] where *P* = (*F*_o_^2^ + 2*F*_c_^2^)/3
*S* = 1.04	(Δ/σ)_max_ = 0.001
8729 reflections	Δρ_max_ = 1.21 e Å^−^^3^
422 parameters	Δρ_min_ = −0.65 e Å^−^^3^
0 restraints	

### Di-µ-hydroxido-bis[chloridodiphenyltin(IV)]–2-ethenylpyridine (1/2) Special details

**Table:**

Geometry. All esds (except the esd in the dihedral angle between two l.s. planes) are estimated using the full covariance matrix. The cell esds are taken into account individually in the estimation of esds in distances, angles and torsion angles; correlations between esds in cell parameters are only used when they are defined by crystal symmetry. An approximate (isotropic) treatment of cell esds is used for estimating esds involving l.s. planes.

### Di-µ-hydroxido-bis[chloridodiphenyltin(IV)]–2-ethenylpyridine (1/2) Fractional atomic coordinates and isotropic or equivalent isotropic displacement parameters (Å^2^)

**Table:**

	*x*	*y*	*z*	*U*_iso_*/*U*_eq_	
Sn1	0.66696 (2)	0.36578 (2)	0.25882 (2)	0.02315 (6)	
Sn2	0.33862 (2)	0.38423 (2)	0.24390 (2)	0.02518 (6)	
Cl1	0.82982 (7)	0.44864 (4)	0.26248 (6)	0.0395 (2)	
Cl2	0.17342 (8)	0.30287 (4)	0.23797 (8)	0.0539 (3)	
O1	0.52597 (18)	0.43077 (9)	0.25227 (12)	0.0254 (4)	
H1	0.5399	0.4750	0.2545	0.059 (8)*	
N1	0.5584 (2)	0.55498 (11)	0.23343 (15)	0.0272 (5)	
C11	0.6352 (3)	0.56690 (15)	0.17342 (19)	0.0327 (7)	
H11	0.6818	0.5331	0.1527	0.047 (4)*	
C12	0.6515 (3)	0.62483 (16)	0.1395 (2)	0.0342 (7)	
H12	0.7060	0.6309	0.0960	0.047 (4)*	
C13	0.5852 (3)	0.67368 (16)	0.1713 (2)	0.0330 (8)	
H13	0.5949	0.7147	0.1507	0.047 (4)*	
C14	0.5046 (3)	0.66270 (15)	0.2335 (2)	0.0303 (7)	
H14	0.4581	0.6960	0.2557	0.047 (4)*	
C15	0.4924 (3)	0.60249 (14)	0.26299 (19)	0.0242 (6)	
C16	0.4087 (3)	0.58645 (17)	0.3284 (2)	0.0327 (8)	
H16	0.4057	0.5440	0.3445	0.047 (4)*	
C17	0.3383 (3)	0.62571 (19)	0.3662 (2)	0.0458 (9)	
H171	0.3385	0.6686	0.3519	0.047 (4)*	
H172	0.2867	0.6114	0.4080	0.047 (4)*	
C31	0.7288 (3)	0.32593 (14)	0.37512 (19)	0.0264 (6)	
C32	0.6575 (3)	0.27954 (15)	0.4104 (2)	0.0311 (7)	
H32	0.5761	0.2676	0.3843	0.042 (5)*	
C33	0.7041 (4)	0.25072 (18)	0.4832 (2)	0.0385 (8)	
H33	0.6547	0.2191	0.5066	0.042 (5)*	
C34	0.8224 (4)	0.26775 (18)	0.5219 (2)	0.0426 (9)	
H34	0.8541	0.2481	0.5720	0.042 (5)*	
C35	0.8939 (3)	0.31337 (18)	0.4875 (2)	0.0428 (9)	
H35	0.9752	0.3250	0.5140	0.042 (5)*	
C36	0.8488 (3)	0.34250 (15)	0.4147 (2)	0.0333 (7)	
H36	0.8993	0.3738	0.3915	0.042 (5)*	
C41	0.7176 (3)	0.32535 (15)	0.1473 (2)	0.0297 (7)	
C42	0.8399 (3)	0.29868 (18)	0.1481 (2)	0.0395 (8)	
H42	0.8994	0.3043	0.1951	0.055 (5)*	
C43	0.8765 (4)	0.2643 (2)	0.0820 (2)	0.0538 (11)	
H43	0.9597	0.2458	0.0839	0.055 (5)*	
C44	0.7905 (4)	0.2570 (2)	0.0134 (2)	0.0577 (12)	
H44	0.8138	0.2325	−0.0317	0.055 (5)*	
C45	0.6706 (4)	0.2853 (2)	0.0097 (2)	0.0504 (10)	
H45	0.6138	0.2818	−0.0390	0.055 (5)*	
C46	0.6331 (3)	0.31876 (18)	0.0767 (2)	0.0386 (8)	
H46	0.5499	0.3372	0.0745	0.055 (5)*	
O2	0.47852 (17)	0.31893 (9)	0.24973 (12)	0.0266 (5)	
H2	0.4626	0.2749	0.2517	0.059 (8)*	
N2	0.4379 (2)	0.19471 (11)	0.26815 (16)	0.0261 (5)	
C21	0.3503 (3)	0.18149 (15)	0.3208 (2)	0.0344 (7)	
H21	0.3005	0.2148	0.3398	0.048 (4)*	
C22	0.3273 (3)	0.12247 (17)	0.3493 (2)	0.0341 (7)	
H22	0.2643	0.1151	0.3874	0.048 (4)*	
C23	0.3997 (3)	0.07448 (16)	0.3203 (2)	0.0339 (8)	
H23	0.3870	0.0330	0.3384	0.048 (4)*	
C24	0.4885 (3)	0.08651 (15)	0.2661 (2)	0.0321 (7)	
H24	0.5380	0.0536	0.2457	0.048 (4)*	
C25	0.5070 (3)	0.14758 (15)	0.24049 (18)	0.0252 (7)	
C26	0.6026 (3)	0.16422 (16)	0.1823 (2)	0.0327 (8)	
H26	0.6075	0.2069	0.1674	0.048 (4)*	
C27	0.6820 (3)	0.1260 (2)	0.1490 (2)	0.0435 (9)	
H27A	0.6809	0.0828	0.1620	0.048 (4)*	
H27B	0.7405	0.1414	0.1119	0.048 (4)*	
C51	0.2903 (3)	0.42367 (14)	0.3567 (2)	0.0290 (7)	
C52	0.3775 (3)	0.42897 (17)	0.4264 (2)	0.0374 (8)	
H52	0.4613	0.4114	0.4261	0.058 (5)*	
C53	0.3422 (4)	0.4598 (2)	0.4960 (2)	0.0490 (10)	
H53	0.4014	0.4625	0.5437	0.058 (5)*	
C54	0.2215 (4)	0.4867 (2)	0.4966 (2)	0.0510 (10)	
H54	0.1985	0.5089	0.5439	0.058 (5)*	
C55	0.1346 (3)	0.4810 (2)	0.4280 (2)	0.0474 (10)	
H55	0.0511	0.4987	0.4285	0.058 (5)*	
C56	0.1680 (3)	0.44974 (17)	0.3589 (2)	0.0363 (8)	
H56	0.1071	0.4459	0.3122	0.058 (5)*	
C61	0.2783 (3)	0.42428 (14)	0.12710 (19)	0.0293 (7)	
C63	0.1134 (4)	0.4390 (2)	0.0153 (2)	0.0478 (10)	
H63	0.0321	0.4279	−0.0117	0.042 (5)*	
C64	0.1856 (4)	0.4849 (2)	−0.0174 (2)	0.0502 (11)	
H64	0.1535	0.5059	−0.0664	0.042 (5)*	
C65	0.3043 (4)	0.50067 (19)	0.0207 (2)	0.0423 (9)	
H65	0.3542	0.5324	−0.0023	0.042 (5)*	
C66	0.3516 (3)	0.47026 (16)	0.0927 (2)	0.0328 (8)	
H66	0.4340	0.4809	0.1184	0.042 (5)*	
C62	0.1583 (3)	0.40882 (17)	0.0871 (2)	0.0379 (8)	
H62	0.1074	0.3773	0.1096	0.042 (5)*	

### Di-µ-hydroxido-bis[chloridodiphenyltin(IV)]–2-ethenylpyridine (1/2) Atomic displacement parameters (Å^2^)

**Table:**

	*U*^11^	*U*^22^	*U*^33^	*U*^12^	*U*^13^	*U*^23^
Sn1	0.01662 (9)	0.01572 (10)	0.03616 (12)	−0.00208 (7)	−0.00347 (8)	0.00538 (8)
Sn2	0.01656 (9)	0.01485 (10)	0.04299 (13)	−0.00109 (7)	−0.00445 (8)	0.00164 (8)
Cl1	0.0240 (4)	0.0234 (4)	0.0689 (6)	−0.0097 (3)	−0.0093 (4)	0.0122 (4)
Cl2	0.0234 (4)	0.0229 (4)	0.1128 (9)	−0.0087 (3)	−0.0107 (5)	0.0067 (5)
O1	0.0190 (9)	0.0128 (10)	0.0433 (13)	−0.0035 (7)	−0.0032 (9)	0.0036 (8)
N1	0.0325 (14)	0.0165 (12)	0.0318 (14)	0.0008 (10)	−0.0019 (11)	−0.0005 (10)
C11	0.0421 (18)	0.0237 (16)	0.0330 (17)	0.0026 (13)	0.0070 (14)	−0.0008 (13)
C12	0.0381 (17)	0.0284 (19)	0.0365 (18)	0.0003 (14)	0.0061 (14)	0.0042 (15)
C13	0.0345 (17)	0.0207 (16)	0.0428 (19)	−0.0021 (13)	−0.0022 (15)	0.0072 (14)
C14	0.0295 (16)	0.0170 (16)	0.0433 (19)	0.0035 (12)	−0.0035 (14)	−0.0007 (13)
C15	0.0222 (14)	0.0169 (14)	0.0324 (16)	0.0006 (11)	−0.0045 (12)	−0.0021 (12)
C16	0.0275 (16)	0.0332 (19)	0.0373 (18)	−0.0017 (13)	0.0025 (14)	0.0034 (14)
C17	0.045 (2)	0.041 (2)	0.054 (2)	0.0002 (17)	0.0208 (18)	0.0020 (19)
C31	0.0263 (15)	0.0192 (15)	0.0335 (16)	0.0046 (12)	0.0015 (13)	−0.0013 (12)
C32	0.0317 (16)	0.0236 (16)	0.0378 (18)	0.0045 (13)	0.0016 (14)	0.0020 (13)
C33	0.046 (2)	0.035 (2)	0.0355 (19)	0.0109 (16)	0.0148 (16)	0.0093 (15)
C34	0.057 (2)	0.046 (2)	0.0241 (17)	0.0239 (18)	−0.0004 (16)	0.0023 (15)
C35	0.0397 (19)	0.048 (2)	0.0386 (19)	0.0128 (17)	−0.0121 (16)	−0.0034 (17)
C36	0.0315 (16)	0.0293 (18)	0.0378 (18)	0.0031 (13)	−0.0044 (14)	−0.0008 (14)
C41	0.0232 (14)	0.0293 (17)	0.0358 (17)	−0.0066 (12)	−0.0018 (13)	0.0079 (13)
C42	0.0270 (16)	0.054 (2)	0.0380 (19)	0.0017 (15)	0.0028 (14)	0.0071 (17)
C43	0.043 (2)	0.073 (3)	0.047 (2)	0.011 (2)	0.0126 (18)	0.006 (2)
C44	0.066 (3)	0.074 (3)	0.035 (2)	0.000 (2)	0.019 (2)	0.003 (2)
C45	0.054 (2)	0.066 (3)	0.0302 (19)	−0.014 (2)	−0.0029 (17)	0.0043 (19)
C46	0.0302 (17)	0.044 (2)	0.040 (2)	−0.0068 (15)	−0.0027 (15)	0.0133 (16)
O2	0.0181 (9)	0.0120 (9)	0.0493 (14)	−0.0038 (7)	0.0003 (9)	0.0026 (8)
N2	0.0282 (13)	0.0137 (12)	0.0365 (14)	−0.0002 (10)	0.0029 (11)	−0.0016 (10)
C21	0.0402 (18)	0.0247 (17)	0.0389 (18)	0.0032 (14)	0.0061 (15)	−0.0005 (14)
C22	0.0357 (17)	0.032 (2)	0.0347 (17)	−0.0005 (14)	0.0053 (14)	0.0037 (15)
C23	0.0397 (18)	0.0185 (16)	0.0423 (19)	−0.0010 (13)	−0.0039 (15)	0.0077 (14)
C24	0.0351 (17)	0.0181 (16)	0.0420 (19)	0.0055 (13)	−0.0030 (15)	−0.0010 (14)
C25	0.0235 (14)	0.0192 (15)	0.0310 (16)	0.0024 (11)	−0.0086 (13)	−0.0028 (12)
C26	0.0314 (17)	0.0279 (18)	0.0385 (19)	0.0029 (13)	0.0004 (14)	0.0003 (14)
C27	0.0396 (19)	0.048 (2)	0.044 (2)	0.0067 (17)	0.0064 (16)	0.0041 (18)
C51	0.0221 (14)	0.0257 (16)	0.0391 (17)	−0.0029 (12)	0.0012 (13)	0.0130 (13)
C52	0.0246 (16)	0.047 (2)	0.0403 (19)	0.0001 (14)	−0.0017 (14)	0.0123 (16)
C53	0.037 (2)	0.075 (3)	0.034 (2)	−0.0112 (19)	−0.0030 (16)	0.010 (2)
C54	0.049 (2)	0.067 (3)	0.039 (2)	−0.007 (2)	0.0142 (18)	0.0025 (19)
C55	0.0308 (18)	0.069 (3)	0.044 (2)	0.0072 (18)	0.0087 (16)	0.0054 (19)
C56	0.0223 (15)	0.047 (2)	0.0398 (19)	0.0009 (14)	0.0016 (14)	0.0081 (16)
C61	0.0289 (15)	0.0239 (16)	0.0339 (17)	0.0094 (13)	−0.0047 (13)	−0.0110 (13)
C63	0.0368 (19)	0.063 (3)	0.041 (2)	0.0229 (19)	−0.0145 (17)	−0.0181 (19)
C64	0.051 (2)	0.069 (3)	0.0297 (19)	0.037 (2)	−0.0031 (17)	−0.0069 (18)
C65	0.050 (2)	0.047 (2)	0.0300 (18)	0.0207 (18)	0.0032 (16)	−0.0012 (16)
C66	0.0349 (17)	0.0319 (18)	0.0307 (17)	0.0084 (14)	−0.0022 (14)	−0.0050 (14)
C62	0.0275 (16)	0.035 (2)	0.049 (2)	0.0108 (14)	−0.0084 (15)	−0.0163 (16)

### Di-µ-hydroxido-bis[chloridodiphenyltin(IV)]–2-ethenylpyridine (1/2) Geometric parameters (Å, º)

**Table:**

Sn1—O1	2.0206 (19)	C44—C45	1.384 (6)
Sn1—C41	2.112 (3)	C44—H44	0.9500
Sn1—C31	2.123 (3)	C45—C46	1.386 (5)
Sn1—O2	2.1974 (18)	C45—H45	0.9500
Sn1—Cl1	2.4538 (7)	C46—H46	0.9500
Sn2—O2	2.0172 (19)	O2—H2	0.9600
Sn2—C51	2.115 (3)	N2—C21	1.330 (4)
Sn2—C61	2.128 (3)	N2—C25	1.340 (4)
Sn2—O1	2.1845 (18)	C21—C22	1.377 (5)
Sn2—Cl2	2.4466 (8)	C21—H21	0.9500
O1—H1	0.9600	C22—C23	1.382 (5)
N1—C11	1.334 (4)	C22—H22	0.9500
N1—C15	1.340 (4)	C23—C24	1.353 (5)
C11—C12	1.376 (4)	C23—H23	0.9500
C11—H11	0.9500	C24—C25	1.393 (5)
C12—C13	1.378 (5)	C24—H24	0.9500
C12—H12	0.9500	C25—C26	1.471 (5)
C13—C14	1.385 (5)	C26—C27	1.312 (5)
C13—H13	0.9500	C26—H26	0.9500
C14—C15	1.388 (4)	C27—H27A	0.9500
C14—H14	0.9500	C27—H27B	0.9500
C15—C16	1.469 (4)	C51—C56	1.392 (4)
C16—C17	1.303 (5)	C51—C52	1.395 (4)
C16—H16	0.9500	C52—C53	1.384 (5)
C17—H171	0.9500	C52—H52	0.9500
C17—H172	0.9500	C53—C54	1.383 (6)
C31—C32	1.393 (4)	C53—H53	0.9500
C31—C36	1.401 (4)	C54—C55	1.380 (5)
C32—C33	1.385 (4)	C54—H54	0.9500
C32—H32	0.9500	C55—C56	1.375 (5)
C33—C34	1.384 (5)	C55—H55	0.9500
C33—H33	0.9500	C56—H56	0.9500
C34—C35	1.376 (5)	C61—C66	1.392 (5)
C34—H34	0.9500	C61—C62	1.398 (4)
C35—C36	1.384 (5)	C63—C64	1.372 (6)
C35—H35	0.9500	C63—C62	1.381 (5)
C36—H36	0.9500	C63—H63	0.9500
C41—C46	1.392 (4)	C64—C65	1.377 (5)
C41—C42	1.395 (4)	C64—H64	0.9500
C42—C43	1.381 (5)	C65—C66	1.392 (5)
C42—H42	0.9500	C65—H65	0.9500
C43—C44	1.378 (6)	C66—H66	0.9500
C43—H43	0.9500	C62—H62	0.9500
			
O1—Sn1—C41	118.04 (10)	C42—C43—H43	120.5
O1—Sn1—C31	119.36 (10)	C43—C44—C45	120.6 (4)
C41—Sn1—C31	121.14 (12)	C43—C44—H44	119.7
O1—Sn1—O2	70.89 (7)	C45—C44—H44	119.7
C41—Sn1—O2	92.22 (9)	C44—C45—C46	120.3 (4)
C31—Sn1—O2	94.50 (10)	C44—C45—H45	119.9
O1—Sn1—Cl1	89.87 (6)	C46—C45—H45	119.9
C41—Sn1—Cl1	95.62 (8)	C45—C46—C41	119.9 (3)
C31—Sn1—Cl1	96.46 (8)	C45—C46—H46	120.0
O2—Sn1—Cl1	160.69 (5)	C41—C46—H46	120.0
O2—Sn2—C51	117.38 (10)	Sn2—O2—Sn1	108.75 (9)
O2—Sn2—C61	118.76 (10)	Sn2—O2—H2	124.0
C51—Sn2—C61	122.37 (11)	Sn1—O2—H2	127.1
O2—Sn2—O1	71.22 (7)	C21—N2—C25	118.0 (3)
C51—Sn2—O1	92.19 (9)	N2—C21—C22	124.0 (3)
C61—Sn2—O1	93.83 (10)	N2—C21—H21	118.0
O2—Sn2—Cl2	90.44 (6)	C22—C21—H21	118.0
C51—Sn2—Cl2	95.91 (8)	C21—C22—C23	117.2 (3)
C61—Sn2—Cl2	95.67 (9)	C21—C22—H22	121.4
O1—Sn2—Cl2	161.66 (6)	C23—C22—H22	121.4
Sn1—O1—Sn2	109.13 (9)	C24—C23—C22	120.0 (3)
Sn1—O1—H1	125.0	C24—C23—H23	120.0
Sn2—O1—H1	125.9	C22—C23—H23	120.0
C11—N1—C15	118.1 (3)	C23—C24—C25	119.5 (3)
N1—C11—C12	124.5 (3)	C23—C24—H24	120.3
N1—C11—H11	117.8	C25—C24—H24	120.3
C12—C11—H11	117.8	N2—C25—C24	121.3 (3)
C11—C12—C13	117.1 (3)	N2—C25—C26	116.2 (3)
C11—C12—H12	121.4	C24—C25—C26	122.5 (3)
C13—C12—H12	121.4	C27—C26—C25	126.5 (3)
C12—C13—C14	119.7 (3)	C27—C26—H26	116.8
C12—C13—H13	120.2	C25—C26—H26	116.8
C14—C13—H13	120.2	C26—C27—H27A	120.0
C13—C14—C15	119.2 (3)	C26—C27—H27B	120.0
C13—C14—H14	120.4	H27A—C27—H27B	120.0
C15—C14—H14	120.4	C56—C51—C52	118.7 (3)
N1—C15—C14	121.4 (3)	C56—C51—Sn2	117.8 (2)
N1—C15—C16	115.6 (3)	C52—C51—Sn2	123.3 (2)
C14—C15—C16	123.0 (3)	C53—C52—C51	120.1 (3)
C17—C16—C15	125.4 (3)	C53—C52—H52	120.0
C17—C16—H16	117.3	C51—C52—H52	120.0
C15—C16—H16	117.3	C54—C53—C52	120.5 (3)
C16—C17—H171	120.0	C54—C53—H53	119.7
C16—C17—H172	120.0	C52—C53—H53	119.7
H171—C17—H172	120.0	C55—C54—C53	119.4 (4)
C32—C31—C36	118.6 (3)	C55—C54—H54	120.3
C32—C31—Sn1	121.4 (2)	C53—C54—H54	120.3
C36—C31—Sn1	119.9 (2)	C56—C55—C54	120.5 (4)
C33—C32—C31	120.6 (3)	C56—C55—H55	119.7
C33—C32—H32	119.7	C54—C55—H55	119.7
C31—C32—H32	119.7	C55—C56—C51	120.7 (3)
C34—C33—C32	120.3 (4)	C55—C56—H56	119.7
C34—C33—H33	119.8	C51—C56—H56	119.7
C32—C33—H33	119.8	C66—C61—C62	118.7 (3)
C35—C34—C33	119.6 (3)	C66—C61—Sn2	120.8 (2)
C35—C34—H34	120.2	C62—C61—Sn2	120.3 (3)
C33—C34—H34	120.2	C64—C63—C62	120.4 (3)
C34—C35—C36	120.8 (3)	C64—C63—H63	119.8
C34—C35—H35	119.6	C62—C63—H63	119.8
C36—C35—H35	119.6	C63—C64—C65	120.2 (4)
C35—C36—C31	120.2 (3)	C63—C64—H64	119.9
C35—C36—H36	119.9	C65—C64—H64	119.9
C31—C36—H36	119.9	C64—C65—C66	120.2 (4)
C46—C41—C42	118.6 (3)	C64—C65—H65	119.9
C46—C41—Sn1	124.2 (2)	C66—C65—H65	119.9
C42—C41—Sn1	117.0 (2)	C65—C66—C61	120.1 (3)
C43—C42—C41	121.5 (3)	C65—C66—H66	119.9
C43—C42—H42	119.2	C61—C66—H66	119.9
C41—C42—H42	119.2	C63—C62—C61	120.5 (4)
C44—C43—C42	119.0 (4)	C63—C62—H62	119.8
C44—C43—H43	120.5	C61—C62—H62	119.8

### Di-µ-hydroxido-bis[chloridodiphenyltin(IV)]–2-ethenylpyridine (1/2) Hydrogen-bond geometry (Å, º)

**Table:**

*D*—H···*A*	*D*—H	H···*A*	*D*···*A*	*D*—H···*A*
O1—H1···N1	0.96	1.76	2.708 (3)	167
O2—H2···N2	0.96	1.76	2.720 (3)	173
